# A nomogram to predict left atrial appendage thrombus and spontaneous echo contrast in non-valvular atrial fibrillation patients

**DOI:** 10.1186/s12872-022-02737-z

**Published:** 2022-07-12

**Authors:** Shikun Sun, Bo Su, Jia Lin, Caiming Zhao, Changsheng Ma

**Affiliations:** Department of Cardiology, The First Affiliated Hospital of Soochow University, Suzhou, 215006 China

**Keywords:** Non-valvular atrial fibrillation, Left atrial appendage thrombus, Left atrial appendage spontaneous echo contrast, Nomogram, Stroke risk

## Abstract

**Background:**

Non-valvular atrial fibrillation (NVAF) significantly increases the risk of stroke. Although there is availability of prediction models, their ability to predict the risk of stroke in NVAF patients remains suboptimal. Therefore, there is need to improve prediction of high-risk individuals, which is critical for efficient management of patients with NVAF.

**Objective:**

The objective of our paper is to develop a nomogram for predicting the risk of left atrial appendage thrombus (LAAT) and spontaneous echo contrast (SEC), thereby replacing the risk of stroke in NVAF patients.

**Design:**

This was a retrospective cohort study that analyzed clinical data and echocardiographic indices of 387 patients with NVAF from October 2018 to June 2021. Multivariable logistic regression was used to evaluate independent factors that were used to construct the prediction nomogram.

**Analysis:**

The discriminative ability and calibration of the nomogram to predict LAAT/SEC were tested using C-statistic and calibration plot. The performance of the nomogram was assessed against the CHA2DS2 score, CHA2DS2-VASc score and ATRIA score using the receiver operating characteristic curve (ROC), decision curve analysis (DCA), integrated discrimination index (IDI) and net reclassification index (NRI).

**Result:**

Out of the total 387 patients enrolled in this study, 232 had LAAT/SEC. Multivariable analyses demonstrated that N-terminal pro-B-type natriuretic peptide (NT-proBNP), albumin (ALB), LAA ejection fraction (LAAEF) and LAA global peak longitudinal strain (LAA GPLS) were independent predictors of LAAT/SEC. The constructed nomogram had good discriminative (C = 0.886) and calibration (0.876) abilities after bias correction by the C-index. Compared with other models, the decision curve analyses demonstrated that the nomogram had greater net benefits. Besides, the nomogram had significant improvement in predictive performance, sensitivity and reclassification for LAAT/SEC compared with the CHA2DS2 [(c-index: 0.886 vs. 0.576, *p* < 0.05), (NRI: 0.539, *p* < 0.05), (IDI: 0.432, *p* < 0.05)], CHA2DS2-VASc [(c-index: 0.886 vs0.579, *p* < 0.05), (NRI: 0.513, *p* < 0.05), (IDI: 0.432, *p* < 0.05)] or ATRIA [(c-index: 0.886 vs0.583, *p* < 0.05), (NRI: 0.546, *p* < 0.05), (IDI: 0.432, *p* < 0.05)].

**Conclusion:**

Taken together, our data demonstrated that the developed nomogram was effective and had potential clinical application in the prediction of LAAT/SEC in patients with NVAF.

## Introduction

Atrial fibrillation (AF) is one of the most common causes of arrhythmia, with a prevalence of as high as 10–17% in people aged 80 years and above [1–3]. Non-valvular atrial fibrillation (NVAF) accounts for most of the AF cases [4]. With acceleration of an ageing global population, there is increased NVAF incidence. The worst consequence of NVAF is thromboembolic stroke. NVAF accounts for 30% of all ischemic strokes, with more than 50% in older patients [5, 6], thus it has been recognized as a serious, global public health challenge. Oral anticoagulation (OAC) is the mainstay preventive treatment option for stroke in patients with NVAF. Previous evidence showed that anticoagulation decreases patients’ risk of ischemic stroke, while increasing the risk of bleeding events or even death [7]. Besides, it remains challenging to accurately identify patients at high risk of stroke, which would guide clinicians in administration of precision therapy.

CHA2DS2 and CHA2DS2-VASc models, which quantify patients’ risk of stroke, are employed to predict the risk of subsequent thromboembolic events in patients with NVAF. However, several studies have demonstrated limited predictive value of these traditional models in risk stratification [8, 9]. Therefore, there is an urgent need to develop new computational methods to predict the risk of stroke in patients with NVAF. Many stroke risk stratification schemes, such as ATRIA score [10], have been proposed. However, most studies are limited to demographic profiling, medical history and serological examination. Due to the deficiency of study design, most models yield limited predictive power. Data has shown that left atrial appendage (LAA) thrombus accounts for up to 90% of NVAF-related strokes, and is used as a surrogate marker of stroke in patients with NVAF [11, 12]. A recent study showed that LAA dysfunction plays an important role in formation of thrombus in LAA [13]. For instance, analysis of LAA strain by speckle tracking echocardiography (STE) reflects the presence of spontaneous echo contrast (SEC) or thrombus in LAA [14]. However, these studies lack data on the role(s) of clinical markers.

With increased analysis of LAA and stroke in NVAF patients, there is need to explore and develop new and accurate prediction models for the assessment of ischemic stroke risk. Left atrial appendage thrombus (LAAT) and LAA-SEC are surrogate indicators of stroke in NVAF patients [15]. Given the significant clinical importance of LAAT/LAA-SEC, we aimed at developing a predictive model for LAAT/SEC in NVAF patients, which would accurately identify patients at high risk of stroke. Besides serological index and demographic data, we defined the indicators of LAA function. Because of the difficulty in discriminating the LAA contractile period, we first applied a new LAA strain parameter which could quantify impaired LAA function, without distinguishing LAA contractile state.

## Methods

### Study population

We enrolled the patients with NVAF who underwent transesophageal echocardiography (TEE) examination at the First Affiliated Hospital of Soochow University between October 2018 and June 2021. Patients were stratified into LAAT/SEC group and SEC/LAAT-free group based on whether they had LAAT/SEC.TEE was performed to confirm existence of LAAT/SEC in the enrolled patients. Patients with clinically relevant cardiac valvular disease, those with primary cardiomyopathies, such as HCM, dilated cardiomyopathy (DCM), unclassified cardiomyopathy (UCM), restrictive cardiomyopathy (RCM), and arrhythmogenic right ventricular cardiomyopathy as well as those with congenital heart disease were excluded from this study. Patients with poor quality images were also not included in this study. Approval for this study was provided by the Ethics Committee of The First Affiliated Hospital of Soochow University (No.: 055/2022).

### Transthoracic echocardiography

All the patients underwent echocardiographic examination (GE, Vivid E9 or GE, Vivid E95 (Norway)), with a 2.5 MHz transducer. From an apical 4-chamber view, we measured right atrial diameter (RAD, the distance from the right atrial lateral wall to the atrial septum at the midpoint of the right atrial length diameter) at end-systole of ventricular. Left atrial diameter (LAD), interventricular septum thickness (IVSth) and left ventricle end-diastolic diameter (LVDD) were measured from the parasternal long-axis view of the heart. The E wave indicates early diastolic mitral inflow velocity when measured by pulsed doppler. The E-wave deceleration time (EDT) was described as the time from the peak of an E wave to the end of an early mitral flow. The left ventricular ejection fraction (LVEF) was assessed using Simpson’s biplane method.

### Transesophageal echocardiography

**Fig. 1:**
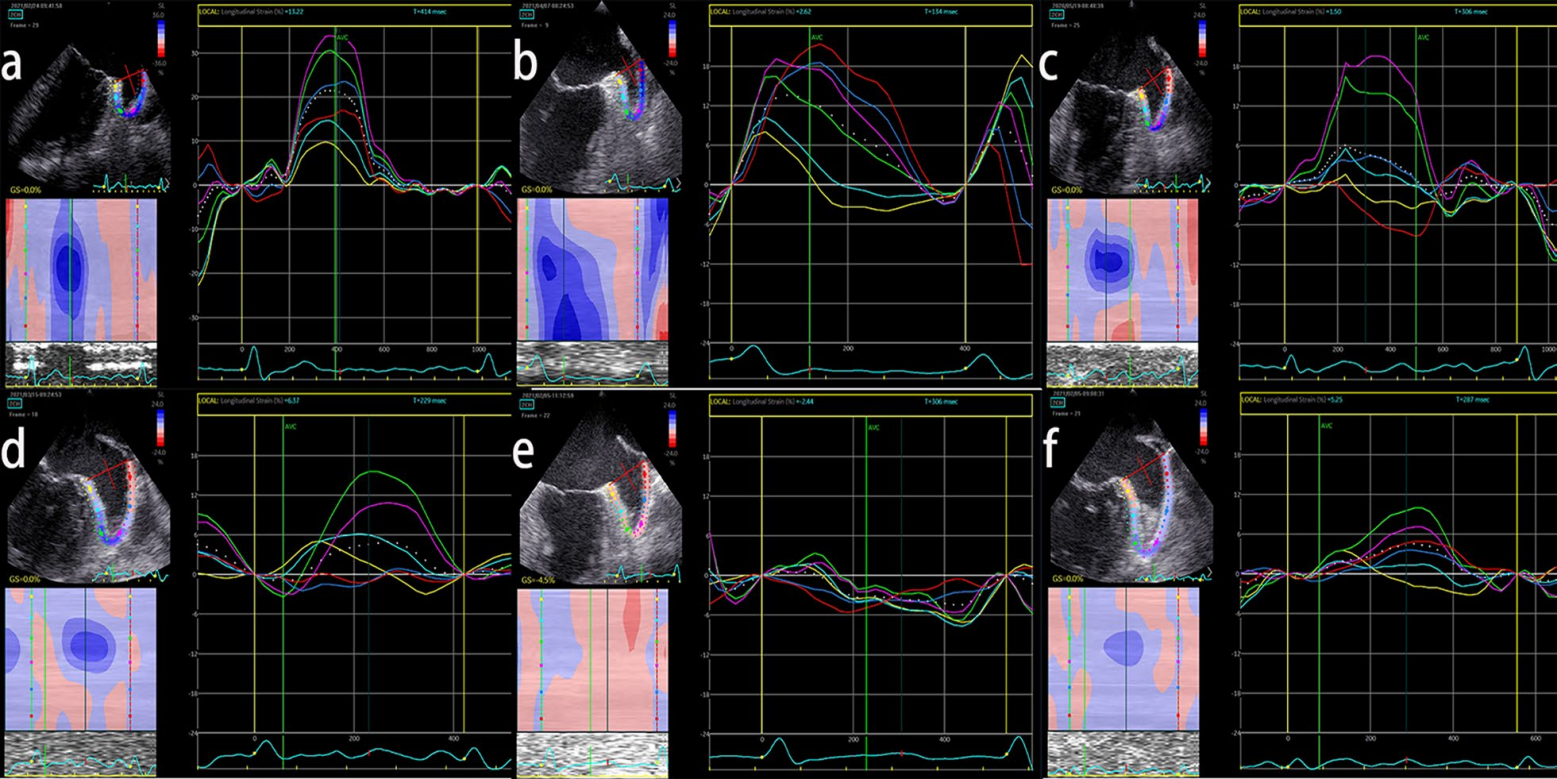
LAA strain curves based on speckle‐tracking in NVAF patients. Images represent the grade of left atrial appendage spontaneous echo contrast (SEC) and left atrial appendage thrombus (LAAT): **a** grade 0, **b** grade 1, **c** grade 2, **d** grade 3, **e** grade 4, **f** LAAT

**Fig. 2:**
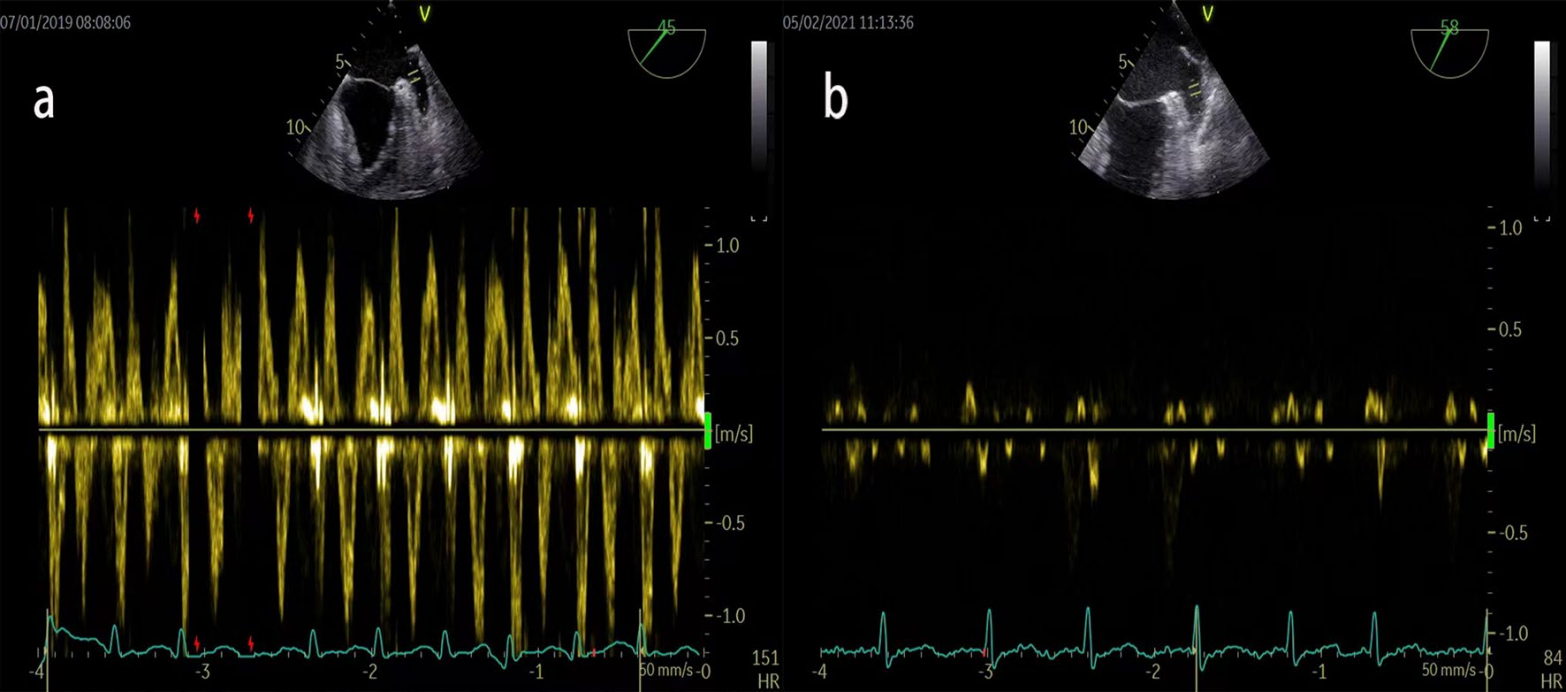
LAA velocity that measured by pulsed wave: **a** patient without left atrial appendage spontaneous echo contrast (SEC); **b** patient with left atrial appendage spontaneous echo contrast (SEC)

The subjects fasted at least 4 h prior to the TEE examination. The long axis view LAA images were visualized in the mid-esophageal view by rotating the imaging sector from 45° to 90°. The LAA ejection fraction (LAAEF) was calculated using the following formula: (maximal LAA volume − minimal LAA volume)/maximal LAA volume] × 100. We defined SEC as cloudy shadows with cyclotron motions within the LAA cavity, which was graded according to Fatkin et al. [15]. Representative cardiac images with LAAT/SEC were shown in Fig. 1. Left atrial appendage diameter (LAAD) was assessed from the circumflex artery to a superior point 1–2 cm inside the left lateral ridge at an angle providing the longest apex to the orifice length. The LAA flow was measured using PW Doppler approximately 1 cm below the LAA cavity outlet after suitable gain and filter adjustment, representative cardiac images with and without LAAT/SEC were shown in Fig. 2.

### Speckle tracking echocardiography

Longitudinal strain analysis was performed offline by two accredited echocardiographers using Echo PAC workstation (GE Healthcare). Longitudinal LAA wall deformation was analyzed by the global peak longitudinal strain. We define the positive global peak strain value minus the negative global peak strain value as the LAA global peak longitudinal strain (LAA GPLS). The LAA GPLS measurements were calculated as an average of 3 heart cycles.

### Clinical characteristics

AF was categorized into paroxysmal or non-paroxysmal following the established protocols. Congestive heart failure was defined as recent heart failure regardless of LVEF or an left ventricular systolic function of moderate to severe reduced on cardiac imaging examinations. The definition of an ischemic stroke was a focal neurological deficit of sudden onset, lasting for more than 24 h, caused by ischemia. TIA was defined as a focal neurologic deficit that appears suddenly, and lasts for 24 h, according to a neurologist. The diagnostic criteria for diabetes, hypertension, embolism and vascular disease were in accordance with latest guidelines or expert consensus [5, 16, 17]. We collected the demographic, clinical and laboratory data of the enrolled patients. Patients who received therapeutic anticoagulation were prescribed for at least 21 days, and the drug dosages followed guidelines. Warfarin-treated subjects needed to maintain an INR between 2 and 3 greater than 21 days. In addition, we calculated the CHA2DS2 score, CHA2DS2-VASc score and ATRIA score for each patient following a previously published criteria [10, 16, 18].

### Statistical analysis

**Fig. 3:**
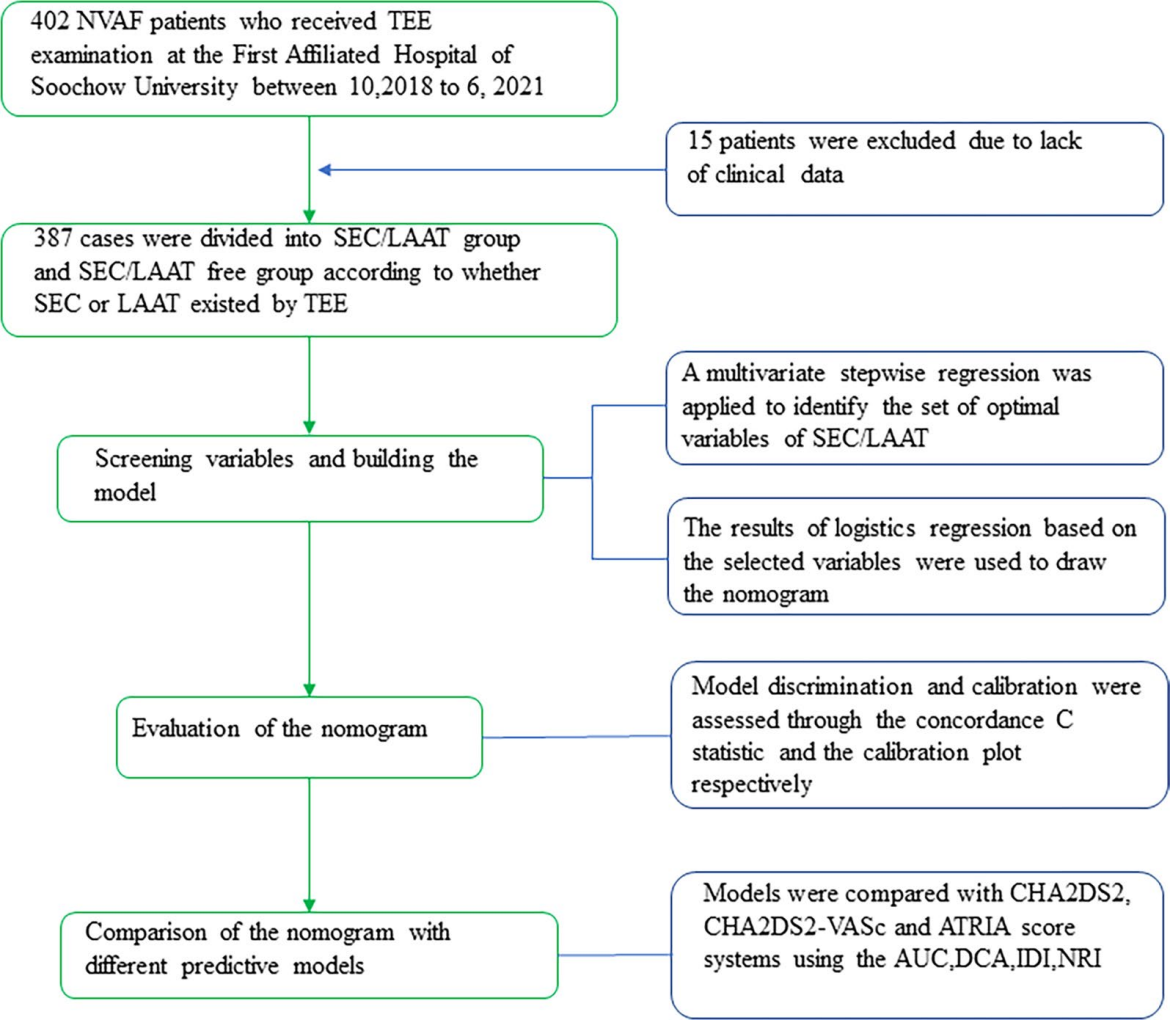
The process of variable selection, model building and evaluation

Normally distributed continuous variables were presented as mean ± standard deviation, while non-normally distributed continuous variables were presented as median (interquartile range). Categorical variables were shown as frequencies (percentages). The difference between two groups was calculated using independent Student’s *t*-test for normal distribution, otherwise, we employed the Mann–Whitney *U* test. For dichotomous categorical variables, a Chi-square test and Fisher’s exact test were performed. An analysis of predictors were carried out using a univariate logistic regression. Accordingly, significant variables of the univariate analyses (*p* < 0.05) were screened using a stepwise regression to solve the multicollinearity problem among variables. Moreover, before using the screened variables to build the prediction model, we conducted collinearity diagnosis, Based on the results of the multivariable analysis of the selected variables, a nomogram was created using the rms package in R, version 4.1 (http://www.r-project.org/). The nomogram was based on proportionally converting each regression coefficient in the multivariable logistic regression to a 0–100 point scale. The effect of the variables with the highest β coefficient was assigned 100 points. The points were added to obtain total points, which were converted to predict the probabilities of LAAT/SEC. We utilized the calibration curves with 1000 bootstrap samples to decrease the overfit bias and validate the calibration of the model. We conducted decision curve analysis (DCA) to define the clinical utility of the model, and utilized DeLong test to evaluate the difference in the performance of our nomogram against other models. The predictive ability of the model was assessed using the concordance C-statistic. In addition, the discriminatory ability of the nomogram was evaluated using the receiver operating characteristic curves (ROC) analysis and the area under the ROC curve (AUC). The predictive performance of the nomogram was compared with other classification models by computing the AUC with the perfcurve function. The added predictive ability of the nomogram was evaluated by integrated discrimination index (IDI) and net reclassification index (NRI). A detailed description of the model construction process can be found in Fig. 3.

## Results

### Clinicopathologic characteristics

**Fig. 4:**
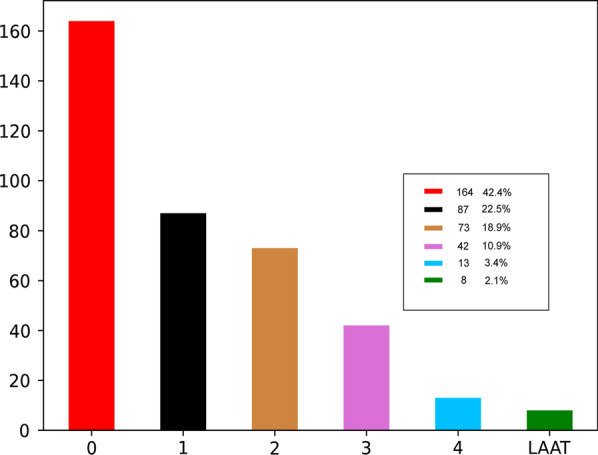
Distribution of LAA SEC/LAAT: 0: grade 0, 1: grade 1, 2: grade 2, 3: grade 3, 4: grade 4. LAAT: left atrial appendage thrombus

402 patients were enrolled in this study. 15 subjects were excluded for lack of clinical data, so, a total of 387 cases were enrolled. Out of the total participants, 232 patients had LAAT/SEC, and the distribution of the subjects in different grades were shown in Fig. 4.

Table1 showed the comparison of demographic, clinical characteristics and echocardiographic findings among the two groups. The proportion of patients with Non-Paroxysmal AF and a history of congestive heart failure was higher in the LAAT/SEC group compared with the SEC/LAAT-free group. The CHA2DS2 score, CHA2DS2-VASc score and ATRIA score were significantly higher in patients with LAAT/SEC compared to those without LAAT/SEC. In addition, the serum level of N-terminal pro-B-type natriuretic peptide (NT-proBNP) was notably higher in the LAAT/SEC group than in the LAAT/SEC-free group. However, there were significantly suppressed albumin levels in the LAAT/SEC group. Echocardiography analysis showed that LAD and RAD were significantly higher in the LAAT/SEC group compared to those in the LAAT/SEC-free group. Moreover, the LVEF values and EDT in patients with LAAT/SEC were significantly lower than those in the LAAT/SEC-free group. Assessment of the LAA function indices demonstrated that the left atrial appendage emptying velocity (LAAEV), left atrial appendage filling velocity (LAAFV), LAAEF, LAAD and LAAGPLS were worse in patients with LAAT/SEC than in the LAAT/SEC-free patients.

**Table 1:** Demographic characteristics and clinical data of the patients

Variables	SEC/LAAT group (n = 223)	SEC/LAAT-free group (n = 164)	*P* value
*Demographics*			
Age (years), median (IQR)	66 (60, 71)	65 (58, 69)	0.06
Age < 65	94 (42.2)	81 (49.4)	
65 ≤ Age < 75 (%)	105 (47.1)	69 (42.1)	
Age ≥ 75 (%)	24 (10.8)	14 (8.5)	
Gender (male, %)	100 (44.8)	98 (59.8)	0.37
BMI (kg/m^2^, mean ± SD)	25.0 ± 4.1	25.2 ± 3.1	0.73
*Medical history*			
Smoking (%)	21 (9.4)	19 (11.6)	0.72
Drinking (%)	38 (17.0)	26 (18.8)	0.10
Non-Paroxysmal AF (%)	168 (75.3)	50 (30.5)	< 0.05
Anticoagulants (%)	103 (46.2)	62 (37.8)	0.08
Warfarin (%)	21 (9.4)	13 (7.9)	
Rivaroxaban (%)	36 (16.1)	22 (13.4)	
Dabigatran (%)	46 (20.6)	29 (17.7)	
*Comorbidities*			
Congestive heart failure (%)	29 (13.0)	4 (2.4)	< 0.05
Hypertension (%)	142 (63.7)	92 (56.1)	0.13
Diabetes mellitus (%)	33 (14.8)	19 (11.6)	0.36
TIA/Stroke/embolism (%)	21 (9.4)	9 (5.5)	0.15
Vascular disease (%)	18 (8.1)	16 (9.8)	0.56
*Predictive models, median (IQR)*			
CHA2DS2 score	2 (1, 3)	2 (1, 3)	0.01
CHA2DS2-VASc score	2 (1, 3)	2 (1, 3)	0.01
0 (%)	20 (9.0)	73 (44.5)	
1 (%)	57 (25.6)	24 (14.6)	
2 (%)	53 (23.8)	49 (29.9)	
≥ 3 (%)	93 (41.7)	18 (11.0)	
ATRIA score	4 (1, 5)	3 (1, 5)	0.01
*Laboratory test, median (IQR)*			
Platelet (10^9^/l)	175.8 ± 53.1	185.4 ± 55.4	0.09
WBC (10^9^/l)	5.8 (4.6, 6.7)	5.5 (4.7, 6.7)	0.66
Hemoglobin (g/l)	141.0 (128.0, 152.0)	140.0 (128.0, 151.0)	0.64
NT-proBNP (pg/ml)	734.2 (313.0, 1447.0)	201.2 (72.7, 462.4)	< 0.05
Positive urine protein (%)	2 (0.9)	4 (2.4)	0.42
Creatinine (umol/L)	68.9 (58.8, 82.5)	67.7 (57.8, 76.5)	0.17
hs-CRP (mg/l)	1.5 (0.7, 3.4)	1.2 (0.6, 2.7)	0.15
ALB (g/l)	38.7 (36.9, 41.3)	40.3 (38.2, 43.0)	< 0.05
D-dimer	0.22 (0.15, 0.35)	0.22 (0.16, 0.35)	0.33
*Echocardiography, median (IQR)*			
LAD (mm)	46.0 (43.0, 51.0)	42.0 (39.0, 45.0)	< 0.05
RAD (mm)	41.0 (37.0, 46.0)	37.0 (35.0, 40.0)	< 0.05
IVSth (mm)	9.0 (9.0, 10.0)	9.0 (9.0, 10.0)	0.58
LVDD (mm)	50.0 (47.0, 53.0)	49.0 (46.0, 52.0)	0.02
LVEF (%)	60.0 (55.0, 63.0)	62.0 (59.0, 65.8)	< 0.05
EDT (ms)	180.0 (148.0, 211.0)	191.5 (157.0, 232.0)	0.01
*LAA function, median (IQR)*			
LAAEV (cm/s)	29.0 (22.0, 42.0)	54.5 (40.0, 71.0)	< 0.05
LAAFV (cm/s)	37.0 (26.0, 51.0)	51.0 (38.0, 67.5)	< 0.05
LAAEF (%)	41.0 (28.0, 59.0)	87.5 (65.3, 95.0)	< 0.05
LAAD (mm)	20.0 (17.0, 23.0)	18.0 (16.0, 21.0)	< 0.05
LAAGPLS	8.9 (6.1, 12.3)	16.6 (13.1, 22.75)	< 0.05

BMI, body mass index; WBC, white blood cell; NT-proBNP, N-terminal pro-B-type natriuretic peptide; hs-CRP, hypersensitive C-reactive protein; ALB, albumin; LAD, left atrial diameter; RAD, right atrial diameter; IVSth, interventricular septum thickness; LVDD, left ventricle end-diastolic diameter; LVEF, left ventricular ejection fraction; EDT, E-wave deceleration time; LAAEV, left atrial appendage emptying velocity; LAAFV, left atrial appendage filling velocity; LAAEF, left atrial appendage ejection fraction; LAAD, left atrial appendage diameter; LAAGPLS, left atrial appendage global peak longitudinal strain

### Multivariable regression analyses and predictive performance

The univariate and multivariable logistic regression model analyses (Table 2) demonstrated that NT-proBNP (OR: 1.01, 95% CI 1.00, 1.01), ALB (0.92, 95% CI 0.86, 1.00), LAAEF (0.97, 95% CI 0.96, 0.98), and LAAGPLS (0.87, 95% CI 0.82, 0.92) were independent predictors for LAAT/SEC.

**Table 2:** Univariate and multivariable logistic regression analyses for LAAT/SEC

	Univariate analysis			Multivariable analysis		
	OR	95% CI	*P*	OR	95% CI	*P*
Non-paroxysmal AF	6.69	4.44, 10.93	< 0.05			
Congestive heart failure	5.98	2.06, 17.37	< 0.05			
CHA2DS2 score	1.27	1.07, 1.50	0.01			
CHA2DS2-VASc score	1.23	1.06, 1.42	0.01			
ATRIA score	1.12	1.03, 1.21	0.01			
NT-proBNP	1.00	1.00, 1.00	< 0.05	1.01	1.00, 1.01	0.012
ALB	0.889	0.84, 0.94	< 0.05	0.92	0.86, 1.0	0.037
LAD	1.14	1.09, 1.19	< 0.05			
RAD	1.12	1.08, 1.17	< 0.05			
LVEF	0.94	0.92, 0.97	< 0.05			
EDT	0.99	0.99, 1.00	< 0.05			
LAAEV	0.96	0.95, 0.97	< 0.05			
LAAFV	0.97	0.96, 0.98	< 0.05			
LAA EF	0.94	0.93, 0.96	< 0.05	0.97	0.96, 0.98	< 0.05
LAAD	1.11	1.05, 1.178	< 0.05			
LAA GPLS	0.79	0.75, 0.83	< 0.05	0.87	0.82, 0.92	< 0.05

AF, atrial fibrillation; ALB, albumin; LAD, left atrial diameter; RAD, right atrial diameter; EDT, E-wave deceleration time; LAAEV, left atrial appendage emptying velocity; LAAFV, left atrial appendage filling velocity; LAAEF, left atrial appendage ejection fraction; LAAD, left atrial appendage diameter; LAAGPLS, left atrial appendage global peak longitudinal strain

### Development and validation of a LAAT/SEC-prediction nomogram

**Fig. 5:**
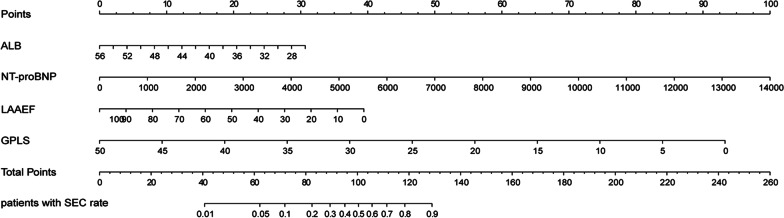
Nomogram for predicting LAAT/SEC

**Fig. 6:**
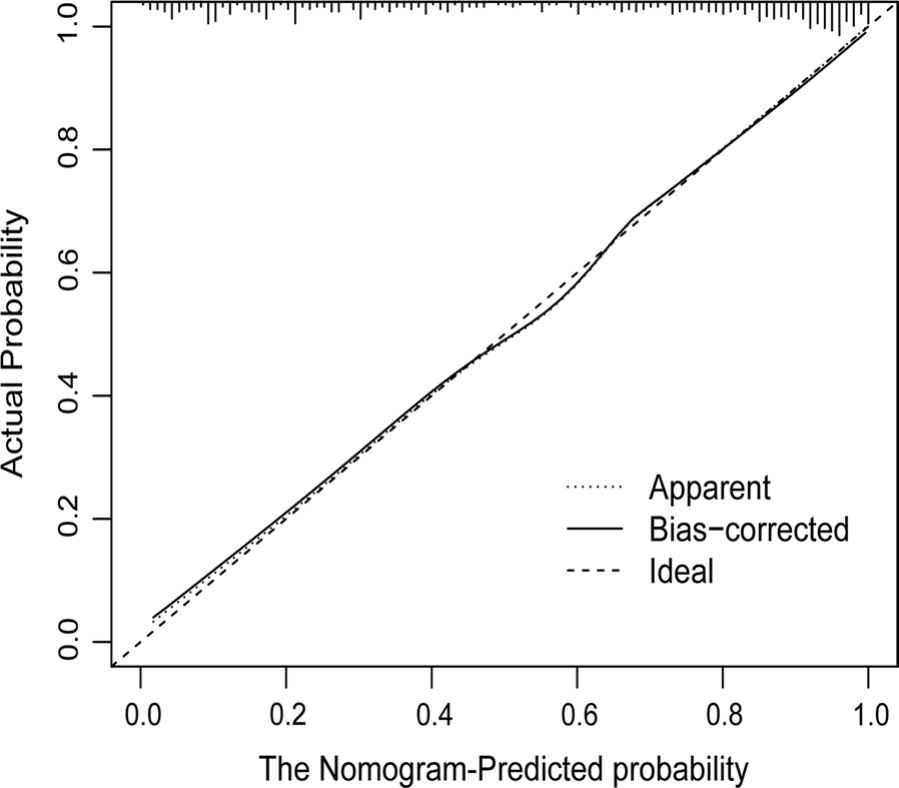
Calibration curves for the nomogram predicting LAAT/SEC. The dotted line represents the performance of the nomogram, while the solid line corrects for any bias in the nomogram

We constructed a nomogram that integrated and quantified the proven independent predictive factors for LAAT/SEC (Fig. 5). We also conducted collinearity diagnosis and the result showed that variance inflation factor (VIF) < 3 means that there is no obvious collinearity between the four variables used to build the prediction model. Our nomogram showed good discrimination efficacy, with a C-statistic of 0.886. To validate the performance of the nomogram, we performed internal validation using bootstrap method with 1000 repetitions. The calibration plot showed that the standard curve of the LAAT/SEC probabilities of the model was very close to the standard 45° diagonal lines, which demonstrated that the new model had good calibration ability (Fig. 6). The analysis showed that the bootstrap C-statistic was 0.876, with a bias of − 0.01.

### Improvement in risk stratification

**Fig. 7:**
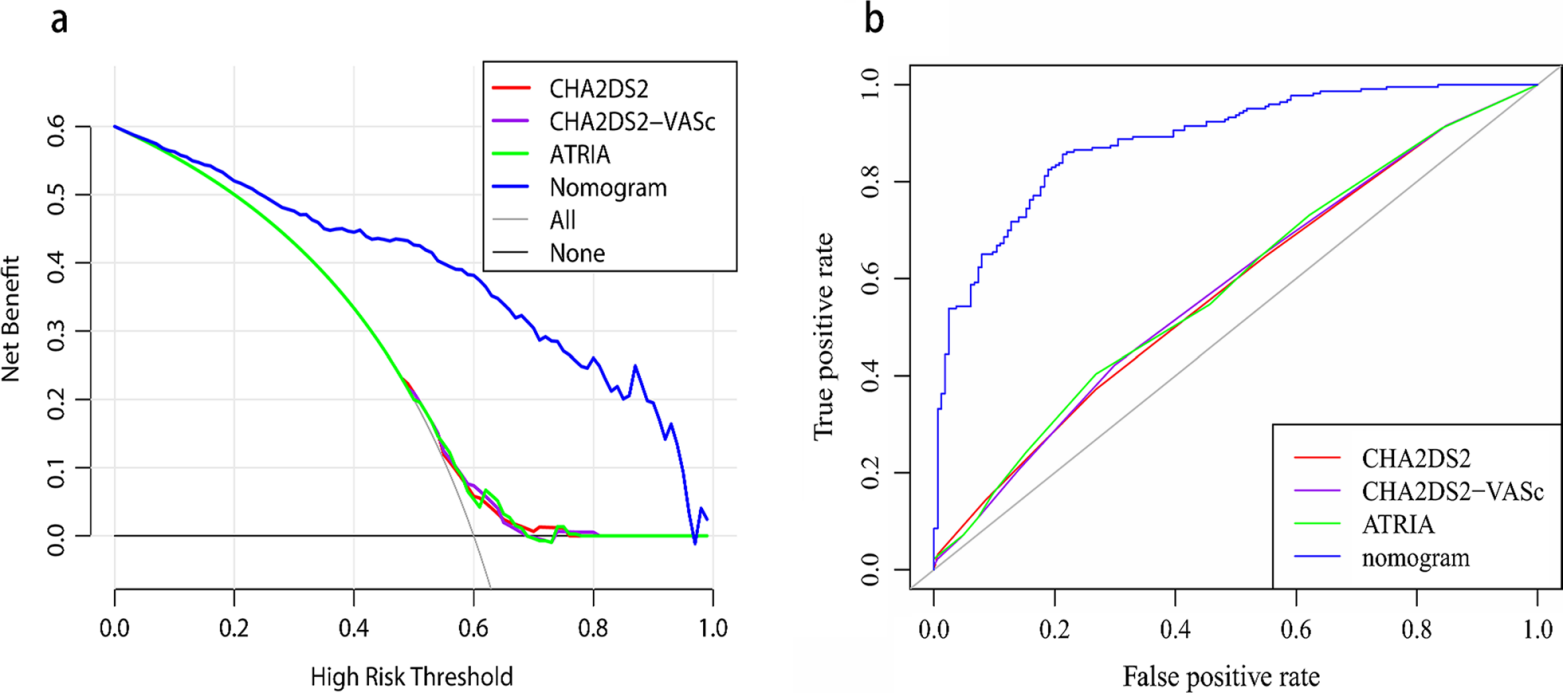
**a** The decision curve plots of the standardized net benefit against different decision thresholds for the four models. The y‐axis represents the net benefit, while the x-axis indicates the range of threshold probabilities. **b** ROC curve comparing the four models

To further evaluate the performance of the nomogram, we compared our model with other three clinically used models. The decision curve showed that our model had increased net benefits compared to the existing models (Fig. 7a). As showed in Fig. 7b, the ROC curve was plotted and the C‐statistic for our model, CHA2DS2, CHA2DS2-VASc and ATRIA was 0.886, 0.576, 0.579 and 0.583, respectively. As shown in Table 3, the NRI for our nomogram compared to the CHA2DS2, CHA2DS2-VASc or ATRIA model was 0.539 (0.416, 0.662, *p* < 0.05), 0.513 (0.391, 0.635, *p* < 0.05), 0.546 (0.422, 0.671, *p* < 0.05), respectively. Besides, our model had improved IDI value compared to CHA2DS2 (0.432, 95% CI 0.381, 0.484, *p* < 0.05), CHA2DS2-VASc (0.432, 95% CI 0.381, 0.484, *p* < 0.05) and ATRIA (0.432, 95% CI 0.381, 0.484, *p* < 0.05).

**Table 3:** Predictive probabilities of the nomogram versus other models

Index	Value	95% CI	*P*
*NRI*			
VS CHA2DS2	0.539	0.416, 0.662	< 0.05
VS CHA2DS2-VASc	0.513	0.391–0.635	< 0.05
VS ATRIA	0.546	0.422–0.671	< 0.05
*IDI*			
VS CHA2DS2	0.432	0.381, 0.484	< 0.05
VS CHA2DS2-VASc	0.432	0.381–0.484	< 0.05
VS ATRIA	0.432	0.381–0.484	< 0.05

NRI, net reclassification improvement; IDI, integrated discrimination improvement

## Discussion

Our study demonstrated that serum albumin is an independent predictor for LAAT/SEC in NVAF patients. We showed that a modified LAA strain was a strong and an independent predictor for LAAT/SEC. Finally, we constructed a combined nomogram that integrates the clinical characteristics, such as the NT-proBNP concentration, the serum ALB level, as well as LAAEF and LAA-GPLS. This study successfully constructed a nomogram based on LAA function that would predict risks of LAAT/SEC.

Moreover, the constructed nomogram could accurately predict the probability of LAAT/SEC and had better performance compared to CHA2DS2, CHA2DS2-VASc or ATRIA scores.

Previous studies showed that LAAT/SEC can occur in about 10–60% of the NVAF patients [15, 19, 20]. Our findings revealed that out of the 387 AF cases, 232 patients had LAAT/SEC. This result was in sync with the findings of a previous study [15]. To date, several serological indicators have been associated with the occurrence and development of LAAT/SEC in NVAF patients. In our study, patients with LAAT/SEC had lower ALB compared to the LAAT/SEC-free patients, thus demonstrating that ALB might be an independent biomarker for prediction of LAAT/SEC. In addition, there is substantial evidence showing that increased risk of thrombosis was invariably associated with a higher level of C-reactive protein [12]. However, our study showed that hs-CRP level was not an important predictor of LAAT/SEC. Previous data has shown that reduction in albumin concentration and the plasma CRP level are inflammatory markers that might result in thrombosis [21–23]. These contradictory findings showed that besides inflammation, ALB was another major contributor to the LAAT/SEC. Another study showed that high concentrations of BNP may correlate with formation of LAAT/SEC in patients with NVAF [15]. It is widely accepted that BNP is a peptide hormone released from LV in response to volume overload and increased pressure. Increased LV end-diastolic pressure in NVAF may lead to increased left atrial (LA) and LAA pressure, which might in turn lead to negative structural remodeling of LA and LAA. The impaired LA and LAA function and consequent blood stagnation of the left atrial appendage may contribute to the development of LAAT/SEC. Similarly, our data showed a higher plasma NT-proBNP level in the LAAT/SEC group.

LAA accounted for approximately 10% of the entire left atrial volume and played an important role in left atrial function. Previous studies [24, 25] have shown that poor contractile function and reduced flow velocity in the LAA were associated with the formation of LAA thrombus. Traditionally, functions of the LAA were mainly assessed by the LAA systole and diastole capability, while the ejection fraction (EF, %) parameter was considered as the main marker of LAA performance and thus widely used to assess the LAA function. A study by Jian Li et al. [13] showed that LAAEF was an independent predictor of thrombus formation in AF patients. In agreement, our study demonstrated that LAAEF was an independent predictor for LAAT/SEC in patients with NVAF.

Myocardial strain derived from speckle tracking imaging is a sensitive parameter for assessment of regional myocardial function and has been widely applied in clinical practice. A previous study demonstrated that LAA strain was an independent factor in the prediction of LAAT [26]. However, limitations such as high complexity and requirement for skilled operators has restricted the uptake of this technology in AF patients. Whereas previous studies focused more on the LAA constriction phase strain, our study used a modified technique-GPLS, and demonstrated a new method for prediction of LAAT/SEC in NVAF patients. We showed that LAAGPLS was a powerful independent predictor for LAAT/SEC in NVAF patients.

Furthermore, to construct a nomogram for precise identification of NVAF patients with SEC and thrombus formation in the LAA, we integrated four independent predictors. Our analyses showed that the developed nomogram had more accurate predictive power compared with the CHA2DS2, CHA2DS2-VASc or ATRIA score system, in identifying patients with LAAT/SEC in NVAF patients. Taken together, the data shows that our nomogram could successfully identify individuals at high risk of NVAF-related stroke, which would guide individualized and accurate treatment strategies for patients with NVAF.

## Study limitations

This study describes preliminary findings based on a single center, with a relatively smaller sample size and limited experimental validation. Besides, patients with co-morbidities and in poorer health were not included due to the invasive procedure.

## Conclusion

Our study analyzed data from 387 patients and identified 4 independent predictors that could predict LAAT/SEC. We successfully developed a nomogram that was able to predict the risks of LAAT/LAA-SEC in NVAF patients.

## Data Availability

Data applied in the course of this study are available from the corresponding author on reasonable request.
